# Whole-Genome Sequencing of a Mycobacterium tuberculosis Strain Belonging to Lineage 1 (Indo-Oceanic) and the East African Indian Spoligotype, Isolated in Jazan, Saudi Arabia

**DOI:** 10.1128/MRA.00717-20

**Published:** 2020-08-13

**Authors:** Aymen Abdelhaleem, Almonther Hershan, Pradeep Agarwal, Abdullah Farasani, Shaheed V. Omar, Arshad Ismail, Mushal Allam

**Affiliations:** a Medical Research Center, Jazan University, Jazan, Saudi Arabia; b Department of Medical Microbiology and Parasitology, College of Medicine, Jeddah University, Jeddah, Saudi Arabia; c Jazan Chest Diseases Hospital, Abu Arish, Saudi Arabia; d Department of Medial Laboratory Technology, Applied Medical College, Jazan University, Jazan, Saudi Arabia; e National Institute for Communicable Diseases, National Health Laboratory Service, Johannesburg, South Africa; f Department of Molecular Medicine and Haematology, School of Pathology, Faculty of Health Sciences, University of Witwatersrand, Johannesburg, South Africa; Portland State University

## Abstract

We describe here the draft genome sequence of AY1MRC, a Mycobacterium tuberculosis strain belonging to lineage 1 (Indo-Oceanic) and the East African Indian spoligotype, isolated from a patient with tuberculosis in Jazan, Saudi Arabia.

## ANNOUNCEMENT

Tuberculosis (TB), for which the etiological agent is the Mycobacterium tuberculosis complex (MTBC), has been considered a global public health emergency since 1931 (1). According to the World Health Organization (WHO), Saudi Arabia has a moderate TB infection rate (2). However, studies have indicated the presence of multidrug-resistant (MDR) and extensively drug-resistant (XDR) TB strains belonging to different lineages circulating in Saudi Arabia in the past 2 decades (3–6). Here, we report the genome sequence of an MTBC strain belonging to the East African Indian spoligotype in lineage 1 (Indo-Oceanic), isolated from a pulmonary TB patient in Jazan, southwest Saudi Arabia.

Mycobacterium tuberculosis strain AY1MRC was isolated from a sputum sample obtained from a 23-year-old male suspected of having pulmonary TB in April 2016. The presence of the bacterium was confirmed through direct smear molecular techniques focused on IS*6110* insertion and *Hsp65* genetic targets, the GeneXpert MTB/RIF assay, and a culture isolated using Lowenstein-Jensen (LJ) medium (7). A single-colony growth of M. tuberculosis complex observed on the LJ medium was used for DNA extraction and purposed for whole-genome sequencing. The genomic DNA was extracted using a Qiagen minikit (Germany) according to the manufacturer’s instructions. The integrity of the extracted DNA was examined by the Genova Plus spectrophotometer (Bibby Scientific, USA) and validated by agarose gel electrophoresis. A paired-end library (2 × 150 bp) was prepared using a TruSeq Nano DNA kit (Illumina, USA) and sequenced on a NovaSeq 6000 instrument (Illumina). The sequencing of AY1MRC produced 22,777,886 raw reads, which were quality trimmed using fastp v0.20.1 (quality [Q], 20 on 90% of bases on each read; read length, 15 bases) (8) and Trimmomatic v0.36 (Q, 20 on 90% of bases on each read; read length, 15 bases) (9), *de novo* assembled using SPAdes v3.13.0 (10) with the parameter “only-error-correction,” and evaluated using QUAST v5.0.2 (11). The assembled genome yielded 43 contiguous sequences longer than 1,000 bp covering 4,386,080 bp, with a G+C content of 65.5%, an *N*_50_ value of 215,026 bp, and a maximum contig length of 395,194 bp. The assembled genome was deposited in GenBank and annotated using the NCBI Prokaryotic Genome Annotation Pipeline (PGAP) (12). Among the 4,144 genes predicted by PGAP, 3,963 were protein-coding genes, 130 were pseudogenes, and 51 were RNAs (3 rRNAs [5S, 16S, and 23S], 45 tRNAs, and 3 noncoding RNAs [ncRNAs]).

The sequencing data were further uploaded to the TB-Profiler webserver (http://tbdr.lshtm.ac.uk/) (13, 14) to predict lineage and drug resistance. AY1MRC was predicted to belong to the Indo-Oceanic lineage (lineage 1.1.2) and wild-type spoligotypes East Africa India 3 and East Africa India 5. Susceptibility to rifampin, isoniazid, aminoglycosides, fluoroquinolones, bedaquiline, delamanid, ethambutol, ethionamide, streptomycin, pyrazinamide, and linezolid was inferred from the genomic data. All software and tools were run with default parameters, unless otherwise indicated.

Ethical clearance was obtained from the Jazan University Standing Committee for Biomedical Research Ethics with approval number 2198/60.

### Data availability.

This whole-genome sequence project has been deposited in DDBJ/ENA/GenBank with the BioProject number PRJNA587526 and BioSample number SAMN12785870 under the accession number WMCK00000000. The described version is WMCK00000000.1. The raw reads have been submitted under the SRA accession number SRR12001309.
